# Clinical implications of novel activating EGFR mutations in malignant peritoneal mesothelioma

**DOI:** 10.1186/1477-7819-8-88

**Published:** 2010-10-13

**Authors:** Jason M Foster, Uppala Radhakrishna, Venkatesh Govindarajan, Joseph H Carreau, Zoran Gatalica, Poonam Sharma, Swapan K Nath, Brian W Loggie

**Affiliations:** 1 Department of Surgery, Creighton Cancer Center, Creighton University, Omaha, NE, USA; 2 Department of Pathology, Creighton University Medical Center, 601 N 30th St., Omaha, NE, USA; 3 Department of Arthritis and Immunology, Oklahoma University, Oklahoma City OK, USA; 4 Department of Surgery, University of Nebraska Medical Center, Omaha, NE, USA

## Abstract

**Background:**

There is a paucity of information about the molecular perturbations involved in MPM tumor formation. We previously reported that EGFR-TK mutations in MPM were predictive of achieving optimal surgical cytoreduction, but the status of EGFR pathway activation potential of these mutations was not known. Here we present the mutant EGFR activating potential and the matured survival data of the EGFR mutant(mut+) relative to wild type EGFR(mut-) mesothelioma.

**Methods:**

Twenty-nine patients were evaluated and their tumors were probed for mutations in the catalytic TK-domain. Twenty-five patients were treated with cytoreductive surgery and complete clinical data was available for comparison of the mut+ and mut- groups. A COS-7 cell expression model was used to determine mutation activating profiles and response to erlotinib.

**Results:**

Functional mutations were found in 31%(9/29) of patients; 7 of these mutations were novel and another was the L858R mutation. All missense mutations were found to be activating mutations and responsive to erlotinib. Of the 25 patients managed surgically, there were 7 mut+ and 18 mut-. Two of 7 (29%) mut+ developed progressive disease and died with a median follow-up time of 22 months; while 13/18 (72%) mut- developed progressive disease and 10/18 (56%) died with median TTP of 12 months and median survival of 14 months.

**Conclusions:**

The novel EGFR mutations identified are activating mutations responsive to erlotinib. The mut+ subset have a 'relative' improved outcome. Erlotinib may have a role in MPM and exploration for mutations in a larger patient cohort is warranted.

## Introduction

Discovering the molecular pathways and mutations active in cancer has resulted in the emergence of novel therapies, as well as, the development of objective predictors of clinical outcome and response to cancer therapies. Perturbations and mutations in the epidermal growth factor receptor gene family have been identified in many cancer subtypes with gain of function alterations occurring at all levels of gene and protein expression [1-4]. Recent studies in non small cell lung cancer (NSCLC) have revealed that mutations in epidermal growth factor receptor (EGFR) occur in 15% of Caucasians and 30% of Asians with NSCLC, and the presence of specific EGFR mutations is predictive of response to therapy and cancer outcome [5-8]. The reported mutations in NSCLC are deletion or missense mutations that occur between exons 18-24 in the tyrosine kinase domain of the receptor. Investigation of EGFR mutations in lung cancers has become a pivotal research paradigm that has begun to unlock the utility of mutations in predicting clinical outcomes, selection of patients for therapies (EGFR-TKIs), and predicting response/resistance to these therapies.

Recapitulation of EGFR mutations in lung cancer cells in vitro have demonstrated that it is an example of an 'oncogenic addiction' mutation which provides a biologic explanation for the improved outcome seen in this EGFR mutant NSCLC group relative to the wild type group[9]. EGFR mutations are likely not limited to lung cancer and pervasive in other cancer. A potential cancer type similar to NSCLC that might harbor functional EGFR mutations is malignant mesothelioma. The first feature common to both malignancies is that EGFR expression is quite common in malignant mesothelioma[10-12]. Also malignant mesothelioma, like NSCLC, is a highly lethal cancer that can arise de novo in the pleural cavity; but unlike NSCLC, mesothelioma can also originate in the peritoneal cavity and soft tissue. Malignant peritoneal mesothelioma (MPM), like pleural mesothelioma, is quite aggressive, with most patients succumbing to this disease within 7-14 months after diagnosis [13]. Treatment of MPM with systemic chemotherapy, radiation, and/or palliative surgery has largely been unsuccessful in improving outcome or extending survival. However, over the last two decades, many groups have shown that aggressive surgical cytoreduction consolidated with intraperitoneal hyperthermic chemotherapy (CRS/IPHC) can improve improved patient outcome in a subset of patients. CRS/IPHC has been the only treatment modality that has yielded long-term survival and cure in selected patients [10,14-19]. At our institution, we perform a high volume of CRS/IPHC for all forms of peritoneal surface malignancies. Through this experience it is evident that many mesothelioma patients do not experience the long term benefits of this aggressive surgical intervention, but all must endure the associated morbidity and mortality. Therefore, identification of surrogate markers that can predict response to CRS/IPHC and lead to novel therapeutic targets in mesothelioma prompted the pursuit of EGFR mutations. We have previously reported that EGFR mutations occur in 31% of MPM, a rate similar to that reported in NSCLC [20]. When mutations were present they correlated with optimal surgical resection in 100% of the patients. Since most patients who present with MPM are unresectable, achieving optimal resection is important because it represents the only reproducible (surrogate) factor that predicts long-term survival [14]. Here we report the first identification of EGFR activating mutations in mesothelioma, as well as, the updated clinical outcome.

## Materials and methods

### Patient population

Twenty-nine consecutive cases of newly diagnosed malignant peritoneal mesothelioma evaluated at Creighton University Medical Center from January 1, 2003 to July 31, 2006 were reviewed. All cases had paraffin embedded tissue available to perform immunostaining and mutation analysis. Institutional review board approval was obtained for this investigation.

### Mutation analysis of *EGFR *in malignant mesothelioma

Tumor tissues were procured from patients who had undergone surgical resection at the department of surgery, Creighton University Medical Center, Omaha, from January 2003 to July 2006. Twenty-nine formalin-fixed paraffin-embedded tissues were available for the analysis. All patients had pathologically proven MPM. The study was approved by the institutional review board of the Creighton University Medical Center. Medical records and hematoxylin and eosin-stained slides of the specimen were reviewed by two pathologists. Tumor tissue with a tumor cell content of greater than 30-40% was chosen for the analysis. In several cases, tissue sections were microdissected manually to obtain both tumor and histologically normal tissue.

Genomic DNA was isolated from tumors embedded in paraffin blocks using Puregene DNA Purification kits (Gentra systems^® ^MN, USA) according to manufacturer's protocol. The *EGFR *gene (Epidermal growth factor receptor, *EGFR*; MIM 131550), (Ensembl Gene ID ENSG00000146648) has a total of 28 exons. Genomic DNA were PCR-amplified for 7 different genomic regions of *EGFR (Exon 18-24) *covering the entire coding sequence of the tyrosine kinase domain and all associated splice junctions. Amplified PCR products were first screened by DHPLC heteroduplex analysis using the Transgenomic WAVE^® ^system (Transgenomic, Omaha, NE) as previously described. Samples with variant DHPLC profiles were purified with QIAquick spin columns and sequenced directly (BigDye^® ^Terminators sequencing kit, Foster City, CA) in both directions using an automated ABI 3100 genetic analysis system and analyzed using the Sequencer 4.1 software program package (Gene Codes^®^, Ann Arbor, MI). Mutant alleles were also cloned by use of the original TA cloning kit (Invitrogen Carlsbad, CA) according to manufacturer's protocol, after PCR amplification, purified and subjected to nucleotide sequencing.

### Functional Analyses of Mutant EGFRs

Full length wild-type *EGFR *cDNA (Gene bank accession No. NM_005228) were cloned into pIRES-hrGFP-2a expression vector (Stratagene^® ^La Jolla, CA). Mutations (W731L, E734Q, T785A, C797Y, Y801H, L831H, L858R and E868G) were introduced into full-length *EGFR *coding sequence by using a QuikChange II XL Site-Directed Mutagenesis kit (Stratagene). All mutant clones were sequenced to ensure that no additional mutations were introduced. The COS-7 cell line was obtained from American Type Culture Collection (ATCC^® ^Manassas, VA) and grown in DMEM with high glucose, 10% FCS, 2 mM L-glutamine (GIBCO^® ^Carlsbad, CA), 10 units/ml penicillin, and 10 μg/ml streptomycin. Cells were transfected (Lipofectamine LXT, Invitrogen) using one tube protocol. Briefly, 12.5 μg of the expression constructs were mixed thoroughly with 2.5 ml of Opti-MEM I reduced serum medium (Invitrogen) and incubated for 30 minutes at room temperature. DNA-Lipofectamine complexes were then added to 8 ml of Opti-MEM I reduced serum medium then plated equal volume in five p60 tissue culture dishes (Falcon^® ^San Jose, CA) and incubated at 37°C and 5% CO_2_. Six hours after the transfection, 2 ml of DMEM with high glucose, 20% FCS was added to each dish. The next day, cells were switched to reduced serum medium and incubated overnight with 10 ng of EGF per milliliter (Sigma^® ^St. Louis, MO). To evaluate the *in vitro *responsiveness of mutant receptors to *EGFR *inhibitor, cells were treated with various concentrations of Erlotinib (Tarceva^® ^LC laboratories, Woburn, MA) three hours before the addition of 10 ng of EGF per milliliter. The Stock solutions of Erlotinib were prepared in DMSO and prior to use, diluted in fresh DMEM media. Three independent experiments were performed for all analyses. Cells were exposed to EGF for 15 minutes. The protein preparation and Western blot analysis was performed following the methods described previously.

Equal amounts of protein were prepared in 50 μl of Laemmli loading buffer and resolved using 4-15% criterion precast Tris-HCL gel electrophoresis (Bio-Rad^® ^Hercules, CA), transferred to nitrocellulose membranes. The efficiency of transfer and uniformity of loading were determined by Ponceau S (Sigma) staining. Western blot analysis was performed with the use of either super signal West femto maximum sensitivity substrate and/or West pico chemiluminescent substrate reagents (Thermo scientific^® ^Rockford, IL). Nitrocellulose membranes were incubated overnight and probed with anti-phospho-*EGFR *(Cell Signaling Technology^®^, Danvers, MA), and *EGFR *antibody (Cell Signaling Technology). The densities of specific protein bands were analyzed by densitometry (Quantity One GS-800 Imaging Densitometer, Bio-Rad^®^).

### Survival and time to progression analysis

The statistical analyses were performed in an exploratory manner on twenty-five patients who underwent surgical exploration for cytoreductive surgery and intraperitoneal hyperthermic chemotherapy. When IPHC was performed, patients received either 30-40 mg of Mitomycin C or 800 mg/M2 of Carboplatin at an inflow temperature of 40.5-42°C for 90-120 minutes. The level of cytoreduction was scored as R1 -- no visible disease, R2a -- residual tumor nodules ≤ 5 mm, R2b -- residual tumor nodules > 5 mm ≤ 2 cm, R2c > 2 cm, and R3-unresectable. Optimal resectability was defined as an R1 or R2a resection. To determine the impact of the presence of mutation on survival and time to progression, a log rank analysis was performed.

## Results

### Mutation analysis

**Figure 1:**
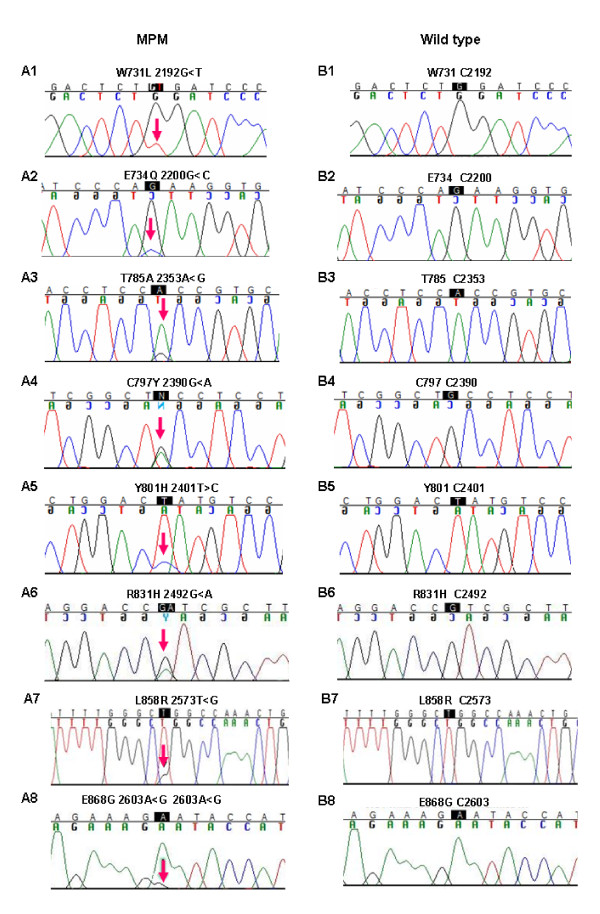
**Identification of missense mutations in MPM tumor samples**. Chromatograms of MPM (Panels A1-A8) and normal (Panels B1-B8) samples.

We analyzed a cohort of MPM samples (n = 29) by DHPLC and sequencing analysis, and identified eight mutations in the tyrosine kinase domain (TKD) of *EGFR*. Of the 8 mutations in the TK domain, 7 were novel (W731L, E734Q, T785A, C797Y, Y801H, L831H and E868G) (Figure 1A, B). One of the mutations (L858R) was previously identified in non-small cell lung cancer (NSCLC) patients and this mutation was found to increase sensitivity to EGFR inhibitor, Erlotinib. All mutations were determined to be somatic, since they were not identified in the analysis of normal tissue from the same patients. Each mutation was only observed once in this cohort, except L831H which was detected in two independent tumors. In addition, analysis of 100 unrelated normal controls from the same ethnic origin did not identify these variants indicating that these are most likely not rare polymorphisms. No mutations were identified within the kinase domain of ERBB2 in this MPM sample set.

EGFR and ERBB2 copy number status was assessed using FISH and quantitative PCR, and no evidence of amplification of either gene was found in all specimens (data not shown). Expression of the EGFR alternative transcript variant, EGFRvIII, could not be detected by RT-PCR (data not shown).

**Figure 2:**
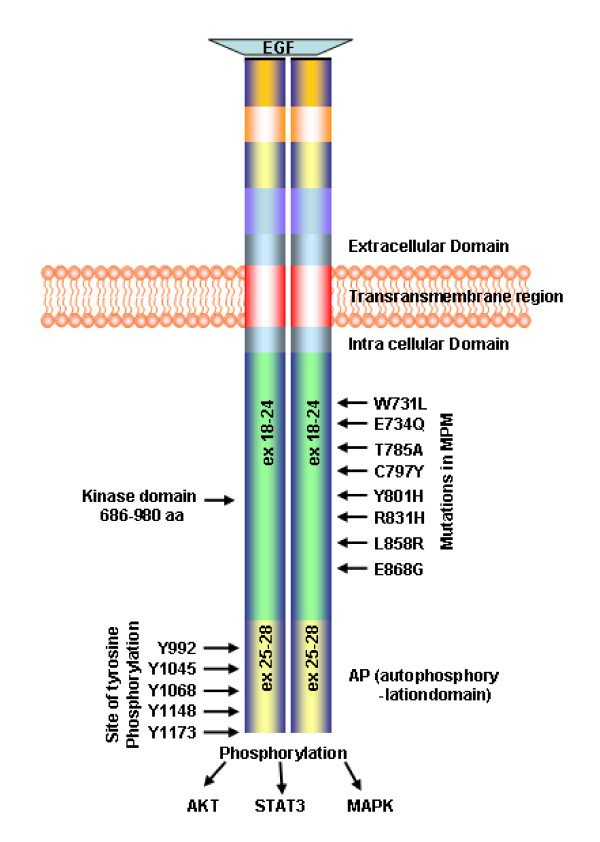
**Schematic representation of *EGFR *protein with the intracellular, transmembrane, extracellular and the TK domains**. The known phosphorylation sites (residues numbered) are marked on the left side. Mutations found in MPM samples are marked on the right. Numbers within bars are exons.

**Figure 3:**
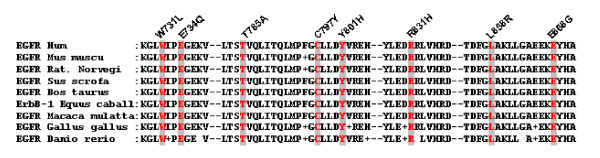
**Amino acid sequence comparisons of *EGFR-TK *domain from members of the *EGFR *family of proteins from different species**. The amino acids at position W731, E734, T785, C797, Y801, R831, L858 and E868 were conserved in several species.

All the mutations were in the TK domain that is critical for *EGFR *activity (Figure 2). Sequence alignment of the human wild- type *EGFR *with the Pfam model of protein kinase domain indicates W731, E734, T785, C797, Y801, R831, L858 and E868 that the mutations were in highly conserved residues (Figure 3).

### Time-response studies of *EGFR *on MM mutations in COS 7 cells

**Figure 4:**
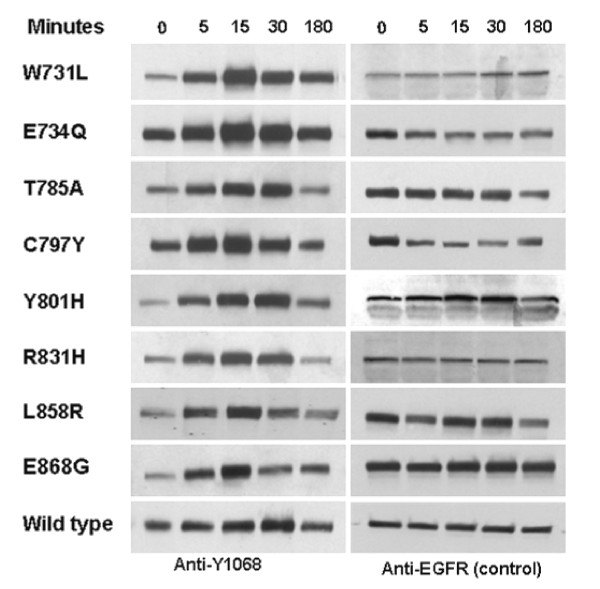
**EGFR mutations from MPM tumor samples are activating mutations**. Western blot analysis of phosphorylated *EGFR *and total *EGFR *from transfectants expressing *EGFR *mutations.

**Figure 5:**
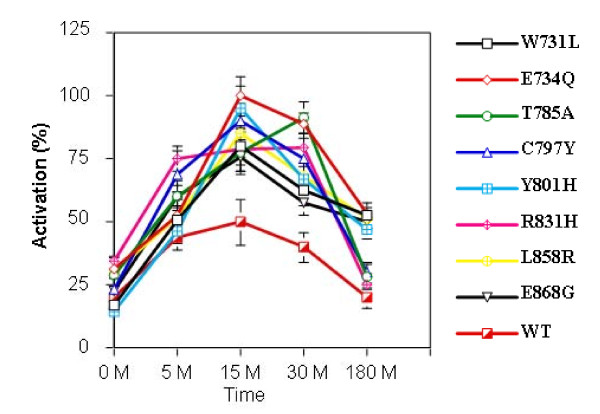
**Quantitative analysis of *EGFR *phosphorylation in COS-7 cell transfectants expressing mutant *EGFR***. Autoradiographs of three independent experiments were quantified. The intensity of EGFR phosphorylation has been adjusted for total EGFR expression. Error bars denote standard deviation.

The functional properties of the *EGFR *mutations were tested by transient transfection assays. The eight mutations were first introduced into the wild type human *EGFR *by site directed mutagenesis. The mutant *EGFRs *were then transfected into COS-7 cells and exposed to *EGF*. Total proteins isolated from these cells were resolved by SDS-PAGE, blotted and probed with anti-phospho *EGFR *(Y1068) and anti-*EGFR *antibodies. *EGFR *activation was assayed by quantification of tyrosine 1068 (Y1068) residue, commonly used as a marker of autophosphorylation of *EGFR *. All mutant *EGFRs *showed enhanced phosphorylation in a time-dependent manner, with a maximal response at 15 minutes. All the 8 mutations showed a similar activation profile (Figure 4, 5). These results demonstrated that the mutations were activating.

### Dose response studies of Erlotinib on COS-7 transfected cells

**Figure 6:**
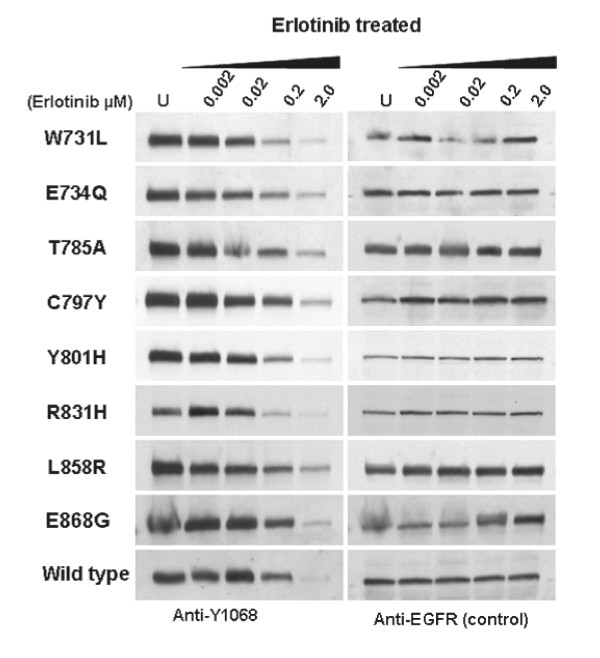
***EGFR *mutations are sensitive to Erlotinib treatment**. Western blot analysis of COS-7 cell lines expressing MPM *EGFR *mutants following treatment with Erlotinib. Inhibition of *EGFR *phosphorylation and total *EGFR *levels were shown relative to controls treated with Erlotinib.

**Figure 7:**
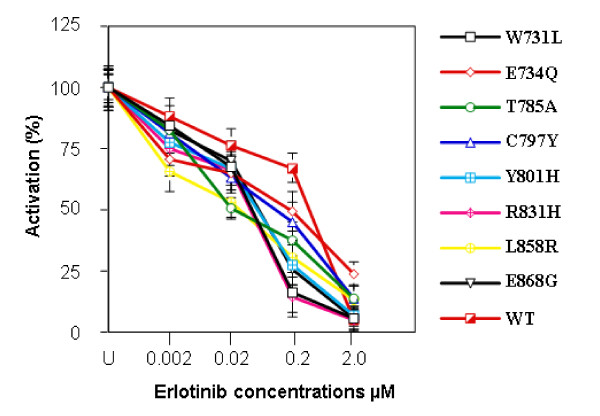
**Quantitative analysis of phosphorylation in COS-7 cell transfectants expressing mutant *EGFR *following treatment with Erlotinib**. Autoradiographs of three independent experiments were quantified. The intensity of EGFR phosphorylation has been adjusted for total EGFR expression. Error bars denote standard deviation.

In order to investigate whether the *EGFR *mutations are sensitive to the EGFR inhibitor Erlotinib, COS-7 cells transfected with mutant EGFR and treated with various concentrations (0.002 to 2.0 μM) of Erlotinib and exposed to EGF. *EGFR *phosphorylation was significantly decreased in a dose-dependent manner for all eight mutants, with a minimum response at .002 μM, a maximal response at 2.0 mM (Figure 6, 7).

### Impact of EGFR mutations on survival and time to progression (TTP)

**Figure 8:**
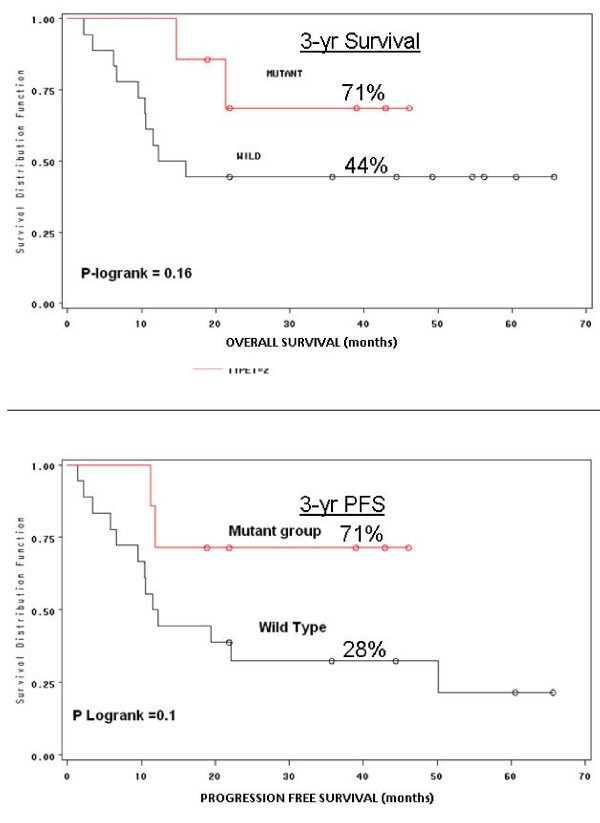
**(A) Log rank analysis of death due to disease and (B) progression free survival based on the presence of a functional EGFR mutation**.

Twenty-five of the 29 patients were treated with surgical cytoreduction and intraperitoneal hyperthermic therapy with median follow-up time of 24 months. Eight patients had tumors with mutant EGFR while the other 17 patients had wild type EGFR. In the EGFR mutant patients, median survival has not been reached but 29% (2/7) of patients have died due to disease progression with a median follow-up time of 22 months; while 56% (10/18) of wild type patients have died due to disease progression with a median survival of 14 months. The time to progression in the wild type group is 12 months with 72% (13/18) of patients developing progressive disease. However, only 29% (2/7) of EGFR mutant patients have developed progressive disease and median TTP has not been reached. Log rank analysis revealed that 3-year OS and DFS was 71% and 71% for the mutant group; 44% and 21% for the wild type group (Figure 8A, B).

## Discussion

We have identified seven novel and one known point mutations in the *EGFR-TK *domain in MPM patients. All mutations are clustered and reside near the ATP-binding cleft of the tyrosine kinase domain. Presence of each mutation in heterozygous condition indicates that these mutant proteins with intact domains may influence wild-type protein expressed from the non-affected allele and/or other *EGFR *factors in a dominant-negative manner by the occupation of their binding sites through mutant *EGFR *proteins. These mutation add to the list of previously identified mutations within the kinase domain of the *EGFR *gene, and also extends the spectrum of malignancies that harbor functional EGFR mutations. To our knowledge, this is the first report identifying functional EGFR mutations in malignant mesothelioma.

Functional analyses of these mutant *EGFRs *in the cultured cells demonstrated that all *EGFR *mutants have enhanced tyrosine kinase activity in response to epidermal growth factor and increased sensitivity to *EGFR *inhibitor Erlotinib. Like other mutants reported in the literature, all eight *EGFR *mutants were ligand dependent and in the absence of EGF stimulation there was little or no activation of any mutant EGFR. These results confirm previous observations showing activation of TKD mutant EGFR as being ligand dependent in similar transient expression systems (NIH3T3 and HeLa cells), while there was no evidence of significant ligand independent EGFR activation.

**Figure 9:**
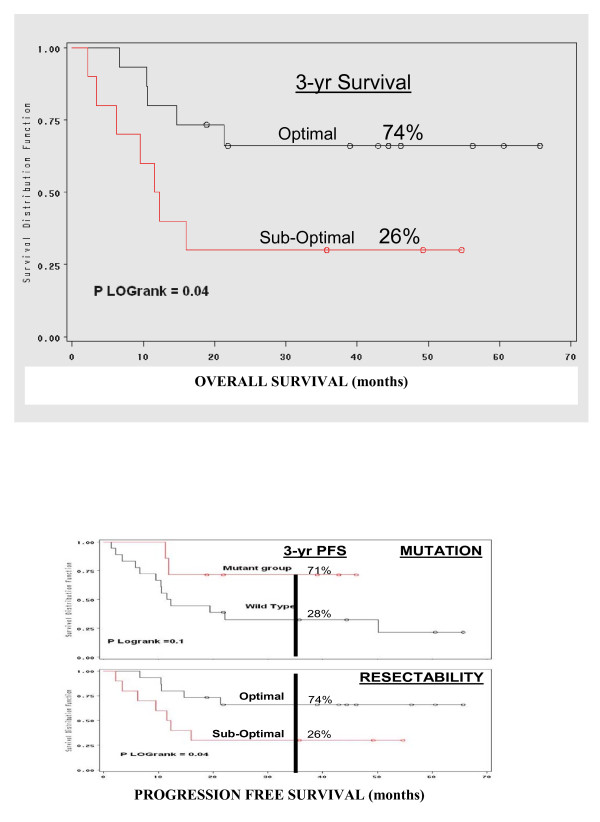
(A) Log rank analysis of death due to disease base on cytoreduction score (optimal vs. sub-optimal, previously published in Annals Surg Oncology 2009 Jan: 16(1) 152-8); (B) Superimposed log rank analysis of death due to disease base on cytoreduction score and PFS based on mutation.

Clinically, the presence of EGFR mutations in MPM appears to predict response to therapy (CRS/IPHC) and represents a potential predictor of improved outcome compared to the wild type MPM. We have previously reported that presence of EGFR mutation(s) in MPM is predictive of optimal resectability, the only reproducible surrogate marker for long term survival with this disease [14]. While resectability is useful, it does not circumvent the associated risks of CRS/IPHC and often cannot be determined preoperatively given the miliary nature of this disease. In figure 9A the optimal resected patients have a statistically significant survival benefit relative to sub-optimal group and a similar outcome was observed in the mutant EGFR patients relative to wild type EGFR group (Figure 9B). In the 13/18 wild type patients that developed progressive disease 10 have died and 3 are currently enrolled in hospice with comfort care measures.

The impact of EGFR mutations in MPM strikingly parallels the observation reported in NSCLC. Specifically the finding that EGFR mutations are predictive of response to therapy [5-8,21-25]. In lung cancer this has been demonstrated in the context of systemic chemotherapy and/or EGFR-TK therapy. In this study, treatment is cytoreductive surgery with intraperitoneal hyperthermic chemotherapy. The finding that EGFR mutations identify responders to therapy mechanistically is explained at the cellular/molecular by the 'oncogenic addition (shock)' model [9,26-28]. In this model, cancers that are dependent on critical oncogenic pathways, like the EGFR pathway for tumor cellular maintenance, undergo an exaggerated/prolonged apoptosis relative to wild type tumor cells when exposed to the same cytotoxic agents [9]. The improved outcome in EGFR MPM mutants is also supported by this model. First, all gross disease is removed surgically, leaving only microscopic to low volume residual disease. This residual disease subsequently is treated with high dose intra-peritoneal chemotherapy and hyperthermia given intra-operatively, and the intrinsic susceptibility of EGFR MPM mutant tumors makes these cells more likely to undergo more extensive apoptosis which manifests as a prolonged progression free survival (Figure 9B).

Although the mutant EGFR group experienced a prolonged survival, two patients in this group have succumbed to their disease, and likely with longer follow-up, more mutant EGFR patients will develop progressive disease. Therefore, the improved outcome observed in the mutant EGFR group is a 'relative' phenomenon. This 'relative' outcome improvement in mutant MPM patients also parallels the observations in metastatic lung cancer which report prolonged median survival but ultimately most patients, including EGFR mutants succumb to progressive diseases. Since NSCLC clinically are responsive to TKI therapy and given the in vitro response of the MPM mutations to Erlotinib, there may be a role for EGFR-TKI therapy in MPM EGFR mutant patients who develop disease recurrence or present with bulky unresectable tumor. Currently EGFR-TKI therapy has not been investigated in peritoneal mesothelioma but a recent trial in pleural mesothelioma did not show any benefit [29]. Interestingly in this erlotinib trial, the high EGFR expressing tumor group experienced a 2-fold longer survival, but study did not interrogate tumors for EGFR mutations. Therefore it is unknown if EGFR mutations occur in pleural mesothelioma or if a subset of mesothelioma patients might benefit from EGFR-TKI therapy and further investigation for perturbations in the EGFR pathway in pleural mesothelioma is warranted.

In summary, we have identified novel activating EGFR mutations in MPM associated with optimal resectability and prolonged survival. Clinically these mutations may ultimately have utility in patient selection for surgery, systemic therapy, and selection for EGFR-TKI. The identification of EGFR mutations in peritoneal mesothelioma expands the spectrum of cancers with EGFR pathway perturbations and provides the first evidence of function EGFR mutations in mesothelioma. Not only does the 'in vitro' biological behavior of these mutations parallel those identified in NSCLC, but the clinical course of MPM patients with EGFR mutant tumors appear to share same 'relative' improved clinical outcome like mutant EGFR-NSCLC. Expanding the cohort of peritoneal mesothelioma and probing for mutations in pleural based disease is warranted.

## Competing interests

The authors declare that they have no competing interests.

## Authors' contributions

JMF participated in study design/conception, clinical data collection/interpretation, coordination, and project oversight. RU performed the basic science analysis that demonstrated mutation activity. VG participated and coordinated the basic science data. JHC performed the patient data extraction for the outcome analysis and validated clinical outcome data. ZG participated in study design, coordination, clinical tissue extraction and analysis resulting in the identification of the EGFR mutations. PS participated in clinical tissue extraction and analysis resulting in the identification of the EGFR mutations. SN performed the statistical analysis. BWL participated in study design, coordination, and oversight. All authors read and approved the final manuscript.
